# Diagnosis of Pentalogy of Cantrell in the First Trimester Using Transvaginal Sonography and Color Doppler

**DOI:** 10.1155/2015/179298

**Published:** 2015-02-23

**Authors:** Ayşe Figen Türkçapar, Ayla Sargın Oruc, Aysegül Öksüzoglu, Nuri Danışman

**Affiliations:** ^1^Obstetrics and Gynecology Department, Bayindir Hospital, 06680 Ankara, Turkey; ^2^Department of Perinatology, Zekai Tahir Burak Women's Health Education and Research Hospital, 06230 Ankara, Turkey

## Abstract

We report the prenatal diagnosis of Cantrell syndrome in the first trimester. During a routine transabdominal ultrasonographic examination, a midline supraumbilical abdominal wall defect including herniated liver and ectopia cordis with a large omphalocele containing the intestines and cystic hygroma was incidentally identified at the 12th week of gestation. A transvaginal sonography examination revealed a severe lumbosacral scoliosis in addition to the inability to visualize the abdominal aorta which was indicative of a severe intracardiac defect. The parents opted for pregnancy to be terminated. In this case report, we discuss the complementary role of transvaginal sonography and Doppler imaging in the diagnosis of Cantrell syndrome in early pregnancy.

## 1. Introduction

Cantrell's syndrome, first described in 1958 [1], is a rare syndrome of congenital defects with a prevalence of 1/65,000–1/200,000 [2], involving a midline anterior ventral wall defect, a defect of anterior diaphragm. a cleft distal sternum, a defect of apical pericardium with communication into the peritoneum, and an intracardiac defect. Only few patients display the full spectrum of anomalies. In 1972, Toyama proposed additional classification of the syndrome: class I, confirmed diagnosis with all five defects present; class II, probable diagnosis with four defects noted (including intracardiac and ventral abdominal wall abnormalities); and class III, incomplete expression when various combinations of defects are present, always including a sternal anomalies [3].

The pathogenesis of Cantrell syndrome is not clear, and the syndrome is considered of heterogeneous origin. Cantrell et al. [1] postulated a developmental failure in differentiation of a segment of the lateral mesoderm around 14 to 18 days of embryonic life. Consequently, the transverse septum of the diaphragm does not develop, and the paired mesodermal folds of the upper abdomen do not migrate ventromedially. Organs may thus eviscerate through the resulting sternal and abdominal wall defects. However, most cases are sporadic and the etiology is still unknown. A few cases associated with trisomy 18 and X-linked inheritance have been described previously [4]. Due to the complexity of the syndrome, the expression of these defects is variable and the prognosis depends on the severity of the lesions. For example, Cantrell syndrome with ectopia cordis (EC) has been associated with a very high perinatal mortality rate characterizing the importance of its differentiation from other abdominal wall defects. The diagnosis is usually made at the beginning of the second trimester. Only a few cases have been confirmed in the first trimester. The differential diagnosis includes isolated thoracic ectopia cordis, amniotic band syndrome, and body stalk anomaly [5].

We report a rare case of Cantrell syndrome diagnosed in the first trimester and discuss the role of transvaginal ultrasonography and Doppler imaging in early gestational period.

## 2. Case Presentation

**Figure 1:**
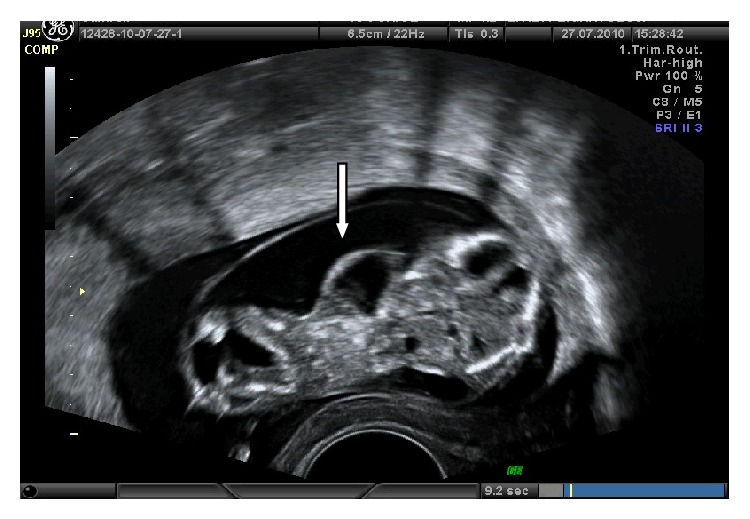
The large omphalocele and herniated liver.

A routine prenatal ultrasound examination in a 22-year-old primigravida was performed for the first trimester screening of aneuploidy. Transabdominal sonographic evaluation using a Voluson 730 Expert scanner (GE Medical systems, Kretztechnik GmbH & OHG, Zipf, Austria) equipped with a 2 to 7 MHz convex transducer demonstrated a live fetus of 12 and 3/7 weeks with a large intact omphalocele containing the intestines. A crown-rump length (CRL) and nuchal translucency measurement could not be obtained due to the cystic hygroma and vertebral deformity. A transvaginal scan using 5 to 9 MHz transvaginal probe of the same sonographic system revealed severe lumbosacral scoliosis and a midline supraumbilical abdominal wall defect including herniated liver and ectopia cordis with a large omphalocele containing the intestines (Figure 1). The abdominal aorta was unable to be visualized which might be indicative of an intracardiac defect.

**Figure 2:**
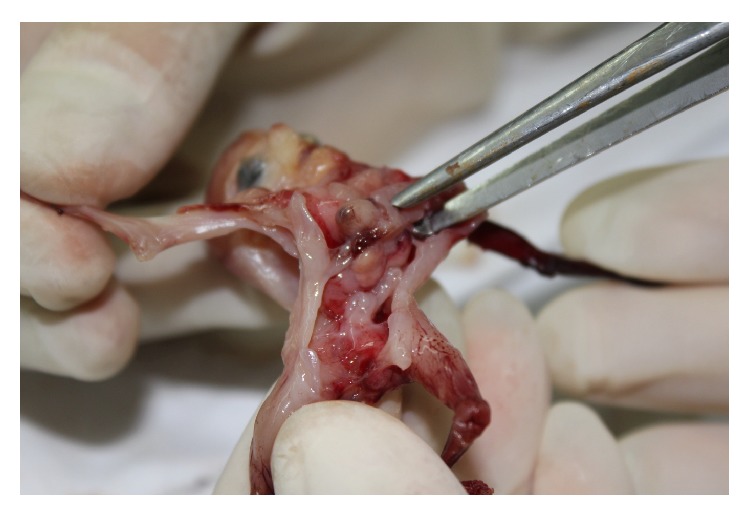
Appearance of the fetus at autopsy.

After counseling the parents about the poor prognosis of the syndrome with complex presentation and multistaged corrective surgical procedures, the parents elected to terminate the pregnancy. The diagnosis was confirmed on the postnatal examination and there were no karyotype anomalies. At the autopsy, a diaphragmatic defect with a large omphalocele, herniated liver, and ectopia cordis (EC) was confirmed. Intracardiac structure was reported to be normal. Postnatal appearance of the fetus is shown in Figure 2.

In this case, we used color Doppler imaging and transvaginal sonography to obtain better images of the defects and increase the precision of our diagnosis in an early gestational age which enabled us to improve our counseling with the parents about the prognosis of the anomalies seen. Informed consent of the parents was obtained for the publication of this case report.

## 3. Discussion

Cantrell syndrome is a rare and complex congenital abnormality occurring as a result of failure in the development of septum transversum [6]. The survival is generally low in this complex syndrome. Multiple corrective surgical procedures are required and the effectiveness, success of treatment, and long-term survival depend primarily on the type and extent of diagnosed anomalies. Cases successfully managed with corrective surgeries have been reported [7–9]. Zidere and Allan reported three cases of Cantrell syndrome whose sonographic findings showed an evolving pattern during gestation and they recommended counselors to be aware of the possibility of an improvement in the ultrasonographic findings in continued pregnancies with this condition [10].

The prenatal diagnosis of Cantrell syndrome in the first trimester was first described by Bennett et al. using 2-dimensional (2D) sonography and Doppler imaging [11]. There are reports that emphasize the role of 3D sonography and fetal MRI in the diagnosis of Cantrell syndrome [2, 12, 13]. Although 3D sonography can provide some additional information especially in vertebral and bone malformations, it is not strictly required for diagnosis. A detailed search for the associated anomalies is mandatory in Cantrell syndrome since the prognosis mostly depends on the severity of these associated findings. McMahon et al. [12] suggested that fetal MRI along with prenatal echocardiography allows optimal assessment of cases with Cantrell syndrome. These modalities may improve our view of prognosis, but they are more crucial for preoperative planning after the first trimester for cases deciding to continue the pregnancy. Intracardiac and various other anomalies like central nervous system and craniofacial abnormalities, polysplenia, and gall bladder agenesis have also been reported in association with this syndrome [14]. A case of Cantrell syndrome associated with the agenesis of fetal limb in a twin pregnancy has been reported recently [15].

In our case, we observed severe lumbosacral lordosis, a large omphalocele, herniated liver, and ectopia cordis. Autopsy of the fetus revealed no intracardiac defects. The existence of ectopia cordis and that of omphalocele are two sonographic findings leading to early prenatal diagnosis of Cantrell syndrome [5]. These defects can be detected by 2D ultrasonography and color Doppler imaging. In cases where the defect is small and difficult to detect, other sonographic markers such as transient pericardial effusion associated with omphalocele are valuable, but they are usually detected in the second trimester [2]. It is well known that the diagnosis of a ventral abdominal defect should be avoided before 12 weeks of gestation, because the embryologic process of elongation of the midgut with herniation into the base of umbilical cord is still in progress in the normal fetus at this time.

In our case, the first sign to draw our attention was the disfiguration of the spinal cord. It was not possible to measure an appropriate CRL and nuchal translucency due to severe lumbar lordoscoliosis. Although a few cases with dorsolumbar scoliosis [16] or scoliosis [17, 18] have been reported, this syndrome is rarely associated with malformations in the vertebral colon. Detection of severe lumbar lordoscoliosis on abdominal ultrasonography led us to a detailed examination for the possible associated defects with a transvaginal approach with color Doppler imaging. Then, we clearly identified the omphalocele and the EC. We consulted the family about the prognosis of the syndrome and multistaged corrective surgical procedures, and the parents opted for pregnancy termination.

In conclusion, a transvaginal ultrasonography provides a better view of the relatively small fetus during the first trimester. Combination of color Doppler further improves the detection of defects and early diagnosis of major anomalies like Cantrell syndrome which would provide timely counseling of the parents.

## References

[1] Cantrell J. R., Haller J. A., Ravitch M. M. (1958). A syndrome of congenital defects involving the abdominal wall, sternum, diaphragm, pericardium, and heart. *Surgery, Gynecology & Obstetrics*.

[2] Desselle C., Herve P., Toutain A., Lardy H., Sembely C., Perrotin F. (2007). Cantrell syndrome: sonographic assesment. *Journal of Clinical Ultrasound*.

[3] Toyama W. M. (1972). Combined congenital defects of the anterior abdominal wall, sternum, diaphragm, pericardium, and heart: a case report and review of the syndrome. *Pediatrics*.

[4] Parvari R., Carmi R., Weissenbach J., Pilia G., Mumm S., Weinstein Y. (1996). Refined genetic mapping of X-linked thoracoabdominal syndrome. *American Journal of Medical Genetics*.

[5] Hsieh Y. Y., Lee C. C., Chang C. C., Tsai H. D., Hsu T. Y., Tsai C. H. (1998). Prenatal sonographic diagnosis of Cantrell's pentalogy with cystic hygroma in the first trimester. *Journal of Clinical Ultrasound*.

[6] Hertzberg B. S., Nyberg D. A., Neilsen I. R., Nyberg D. A., Mc Gahan J. P., Pretorius D. H., Pilu G. (2003). Ventral wall defects. *Diagnostic Imaging of Fetal Anomalies*.

[7] Vazquez-Jimenez J. F., Muehler E. G., Daebritz S. (1998). Cantrell's syndrome: a challenge to the surgeon. *Annals of Thoracic Surgery*.

[8] Grethel E. J., Hornberger L. K., Farmer D. L. (2007). Prenatal and postnatal management of a patient with Cantrell syndrome and left ventricular aneurysm. A case report and literature review. *Fetal Diagnosis and Therapy*.

[9] Takaya J., Kitamura N., Tsuji K. (2008). Pentalogy of Cantrell with a double-outlet right ventricle: 3.5-year follow-up in a prenatally diagnosed patient. *European Journal of Pediatrics*.

[10] Zidere V., Allan L. D. (2008). Changing findings in pentalogy of cantrell in fetal life. *Ultrasound in Obstetrics and Gynecology*.

[11] Bennett T. L., Burlbaw J., Drake C. K., Finley B. E. (1991). Diagnosis of ectopia cordis at 12 weeks gestation using transabdominal ultrasonography with color flow Doppler. *Journal of Ultrasound in Medicine*.

[12] McMahon C. J., Taylor M. D., Cassady C. I., Olutoye O. O., Bezold L. I. (2007). Diagnosis of pentalogy of Cantrell in the fetus using magnetic resonance imaging and ultrasound. *Pediatric Cardiology*.

[13] Gün Ï., Kurdoğlu M., Müngen E., Muhcu M., Babacan A., Atay V. (2010). Prenatal diagnosis of vertebral deformities associated with pentalogy of Cantrell: the role of three-dimensional sonography?. *Journal of Clinical Ultrasound*.

[14] Peixoto-Filho F. M., do Cima L. C., Nakamura-Pereira M. (2009). Prenatal diagnosis of pentalogy of cantrell in the first trimester: is 3-dimensional sonography needed?. *Journal of Clinical Ultrasound*.

[15] Cakiroglu Y., Doger E., Yildirim Kopuk S., Babaoglu K., Caliskan E., Yucesoy G. (2014). Prenatal diagnosis of Cantrell’s Pentalogy associated with agenesis of left limb in a twin pregnancy. *Case Reports in Obstetrics and Gynecology*.

[16] Nanda S., Agarwal U., Sen J., Sangwan K. (2003). Cantrell's syndrome—report of two cases with one atypical variant. *Archives of Gynecology and Obstetrics*.

[17] Aslan A., Karagüzel G., Ünal I., Aksoy N., Melikoglu M. (2004). Two rare cases of the Cantrell syndrome or its variants. *Acta Medica Austriaca*.

[18] van Hoorn J. H. L., Moonen R. M. J., Huysentruyt C. J. R., van Heurn L. W. E., Offermans J. P. M., Mulder A. L. M. T. (2008). Pentalogy of Cantrell: two patients and a review to determine prognostic factors for optimal approach. *European Journal of Pediatrics*.

